# Only one of the two type VI secretion systems encoded in the *Salmonella enterica* serotype Dublin genome is involved in colonization of the avian and murine hosts

**DOI:** 10.1186/1297-9716-45-2

**Published:** 2014-01-09

**Authors:** David Pezoa, Carlos J Blondel, Cecilia A Silva, Hee-Jeong Yang, Helene Andrews-Polymenis, Carlos A Santiviago, Inés Contreras

**Affiliations:** 1 Departamento de Bioquímica y Biología Molecular, Facultad de Ciencias Químicas y Farmacéuticas, Universidad de Chile, Santos Dumont 964, Santiago, Chile; 2 Department of Microbial and Molecular Pathogenesis, College of Medicine, Texas A&M University System Health Science Center, 407 Joe H. Reynolds Medical Building, College Station, TX 77843-1114, USA

## Abstract

The type VI secretion system (T6SS) is a virulence factor for many Gram-negative bacteria. S*almonella* genus harbors five phylogenetically distinct T6SS loci encoded in *Salmonella* Pathogenicity Islands (SPIs) SPI-6, SPI-19, SPI-20, SPI-21 and SPI-22, which are differentially distributed among serotypes. The T6SSs encoded in SPI-6 and SPI-19 contribute to pathogenesis of serotypes Typhimurium and Gallinarum in mice and chickens, respectively. *Salmonella* Dublin is a pathogen restricted to cattle where it causes a systemic disease. Also, it can colonize other hosts such as chickens and mice, which can act as reservoirs of this serotype. *Salmonella* Dublin harbors the genes for both T6SS_SPI-6_ and T6SS_SPI-19_. This study has determined the contribution of T6SS_SPI-6_ and T6SS_SPI-19_ to host-colonization by *Salmonella* Dublin using avian and murine models of infection. Competitive index experiments showed that, a mutant strain lacking both T6SSs (∆T6SS_SPI-6_/∆T6SS_SPI-19_) presents a strong colonization defect in cecum of chickens, similar to the defect observed for the ∆T6SS_SPI-6_ mutant, suggesting that this serotype requires a functional T6SS_SPI-6_ for efficient colonization of the avian gastrointestinal tract. Colonization of mice was also defective, although to a lesser extent than in chickens. In contrast, the T6SS_SPI-19_ was not necessary for colonization of either chickens or mice. Transfer of T6SS_SPI-6_, but not T6SS_SPI-19_, restored the ability of the double mutant to colonize both animal hosts. Our data indicate that *Salmonella* Dublin requires only the T6SS_SPI-6_ for efficient colonization of mice and chickens, and that the T6SS_SPI-6_ and T6SS_SPI-19_ are not functionally redundant.

## Introduction

The genus *Salmonella* contains over 2500 serotypes distributed in two species, *S. enterica* and *S. bongori*[1]. Depending on the serotype and the immune status of the infected host, *Salmonella* can produce a wide spectrum of clinical signs ranging from self-limiting diarrhea to systemic illness. Some serotypes are able to infect a wide range of hosts, while others can infect only one animal species [2]. *Salmonella enterica* serotype Dublin (*Salmonella* Dublin) infection is restricted to cattle, where it causes a serious systemic disease characterized by pneumonia, arthritis, osteomielytis, meningoencephalitis, enteritis and, in some cases, abortion, leading to economic losses in animal industry [3-7]. *Salmonella* Dublin can also infect humans via consumption of raw milk from infected cattle, constituting an important threat to public health [8-10]. In addition, some studies have reported the isolation of *Salmonella* Dublin from chickens and wild mice, suggesting that these animal hosts can act as environmental reservoirs of *Salmonella Dublin*. In one report, *Salmonella* Dublin was isolated from feces of wild mice in a farm with high prevalence of this pathogen in cattle, suggesting that rodents may act as reservoirs and eventually contribute to *Salmonella* Dublin transmission to susceptible cows [11]. Chickens may also act as reservoirs for this serotype, as *Salmonella* Dublin is able to colonize chickens without clinical symptoms, contributing to transmission of salmonellosis to cattle and humans [12].

The type VI secretion system (T6SS) represents a new paradigm of protein secretion and is a key virulence factor for many important pathogenic bacteria contributing to different processes ranging from inter-bacterial killing to pathogenesis [13-17]. The genus S*almonella* contains five phylogenetically distinct T6SS loci encoded in differentially distributed *Salmonella* Pathogenicity Islands (SPIs) (i.e. SPI-6, SPI-19, SPI-20, SPI-21 and SPI-22) [18,19]. Some serotypes contain a unique T6SS, while others have two different T6SS loci encoded in their genomes. Whether the presence of multiple T6SSs corresponds to differential functional specialization of each system, or if they are redundant in their function is currently unknown. Interestingly, the genome of *Salmonella* Dublin includes two T6SSs encoded in SPI-6 and SPI-19, respectively.

Recent reports have linked these T6SSs to *Salmonella* virulence and colonization. The T6SS encoded in SPI-6 (T6SS_SPI-6_) is required by *Salmonella* Typhimurium for intracellular survival in avian and murine macrophages and for gastrointestinal colonization and systemic spread in orally-infected White Leghorn chicks and BALB/c mice [20-22]. In addition, transposon insertions in genes encoding essential components of T6SS_SPI-6_ in *Salmonella* Typhi produced an attenuated phenotype in a novel humanized mice model of infection [23]. On the other hand, the T6SS encoded in SPI-19 (T6SS_SPI-19_) contributes to the intracellular survival of *Salmonella* Gallinarum in avian macrophages [24], and to the gastrointestinal and systemic colonization of infected chicks by this serotype [25]. These findings have supported the notion that T6SSs could be functionally redundant despite distinct phylogenetic origins [21,25]. In this context, *Salmonella* Dublin constitutes a suitable model to study the contribution of two differentially-encoded T6SSs to host-adaptation and pathogenesis of *Salmonella*.

We evaluated the contribution of T6SS_SPI-6_ and T6SS_SPI-19_ to the colonization of the gastrointestinal tract and deeper tissues by *Salmonella* Dublin using avian and murine models of infection. A strain of *Salmonella* Dublin lacking both T6SS_SPI-6_ and T6SS_SPI-19_ displayed a strong colonization defect of the cecum, liver and spleen in competitive infections in both animal models. Furthermore, we observed a similar phenotype in a strain that lacks only the T6SS_SPI-6_. Interestingly, this colonization defect could be reversed by transfer of a complete T6SS_SPI-6_, but not by transfer of the T6SS_SPI-19_. These results suggest that T6SS_SPI-6_ and T6SS_SPI-19_ are not functionally redundant in *Salmonella* Dublin, and that only T6SS_SPI-6_ is required for host colonization by this serotype.

## Material and methods

### Bacterial strains and growth conditions

The bacterial strains used in this study are listed in Table 1. Bacteria were routinely grown in Luria-Bertani (LB) broth (10 g/L tryptone, 5 g/L yeast extract, 5 g/L NaCl) at 37 °C with aeration. LB broth was supplemented with ampicillin (Amp; 100 μg/mL), kanamycin (Kan; 50 μg/mL), chloramphenicol (Cam; 20 μg/mL), trimethoprim (Tp; 100 μg/mL), or spectinomycin (Sp; 250 μg/mL) as needed. LB plates were solidified by the addition of agar (15 g/L) to LB broth.

**Table 1:** Strains and plasmids used in this study

**Strains**	**Features**	**Source of reference**
***Escherichia coli***		
DH5α	F^-^Φ80*lacZ*∆M15∆(*lacZYA-argF*)U169 *deoR recA1 endA1 hsdR17*(r_k_^-^, m_k_^+^) *phoA supE44 thi-1 gyrA96 relA1* λ^-^	Laboratory collection
EC100D *pir-116*	*F*^*-*^*mcrA∆(mrr-hsdRMS-mcrBC)*Φ *80dlacZ∆M15 ∆lacX74 recA1 endA1 araD139 ∆(ara, leu)7697 galU galK λ- rpsL (Str*^*R*^*) nupG pir-116(DHFR)*	Laboratory collection
EC100D *pir-116*/R995 + SPI-6	Strain carrying the T6SS_SPI-6_ from *S*. Typhimurium cloned in plasmid R995	This study
EC100D *pir-116*/R995 + SPI-19	Strain carrying the T6SS_SPI-19_ from *S*. Gallinarum cloned in plasmid R995	[25]
DH5α/R995	Strain harboring an empty R995 vector	This study
DH5α/R995-VC6	Strain harboring plasmid R995-VC6	This study
***Salmonella *** **Dublin**		
CT_02021853	Wild-type strain	Laboratory collection
MSD753	CT_02021853 ∆*phoN*::FRT	This study
MSD35	CT_02021853 ∆T6SS_SPI-6_ ∆T6SS_SPI-19_	This study
MSD36	CT_02021853 ∆T6SS_SPI-6_	This study
MSD37	CT_02021853 ∆T6SS_SPI-19_	This study
WT/R995	CT_02021853 containing an empty R995 vector	This study
MSD35R	MSD35 harboring R995 plasmid	This study
MSD35R6	MSD35 complemented with plasmid R995 + SPI-6	This study
MSD35R19	MSD35 complemented with plasmid R995 + SPI-19	This study
**Plasmids**		
pKD46	*bla* P_BAD_*bet exo* pSC101 oriT^s^, Amp^R^	[26]
pCLF2	Red-swap redesigned vector, Cam^R^	[27]
pCLF4	Red-swap redesigned vector, Kan^R^	[27]
pEKA30	IncQ plasmid that constitutively express Cre recombinase, Amp^R^	[28]
pVEX1212	Suicide vector harboring a *loxP* site followed by a Sp^R^ cassette	[28]
pVEX2212	Suicide vector harboring a *loxP* site followed by a Cam^R^ cassette	[28]
R995	Self-transmissible broad-host range IncP vector	[28]
R995-VC6	A derivative of plasmid R995 with a cloned 1,209 bp DNA fragment of T6SS_SPI-6_ from *S*. Dublin	This study
R995 + SPI-6	T6SS_SPI-6_ cluster from *S*. Dublin cloned in vector R995	This study
R995 + SPI-19	T6SS_SPI-19_ cluster from *S*. Gallinarum 287/91 cloned in vector R995	[25]

### DNA procedures and PCR amplifications

DNA manipulations were performed using standard protocols. Plasmid DNA was isolated using the “QIAprep Spin Miniprep Kit” (QIAGEN, MD, USA). Genomic DNA was isolated using the “GenElute Bacterial Genomic DNA” kit (Sigma-Aldrich, MO, USA). PCR products were purified using the “QIAquick PCR Purification Kit” (QIAGEN, MD, USA). Ligations were performed using T4 DNA ligase (NEB, MA, USA) as recommended by the manufacturer. DNA samples were analyzed by electrophoresis in 1% agarose gels and were visualized under UV light after RedGel (Biotium, CA, USA) staining.

Primers were designed using the “Vector NTI Advance 10.0” software (Invitrogen, CA, USA) and are listed in Table 2. PCR products were amplified in a “MultiGene TC9600-G” thermal cycler (LabNet, NJ, USA). PCR reaction mixes contained 1X buffer, 2 mM MgCl_2_, 100 nM dNTPs, 100 nM of each primer, 100 ng of template DNA and 0.5 to 1 U of HiFi DNA pol (KAPA, MA, USA). Standard conditions for amplification were: 2 min at 95 °C, followed by 30–35 cycles of 94 °C for 45 s, 55 °C for 30 s and 72 °C for a suitable time (1 min/kb) according to DNA polymerase processivity, and a final extension step at 72 °C for 5 min.

**Table 2:** Primers used in this study

**Primer**	**Sequence**^***a***^
**Mutagenesis**	
SPI-6_T6SS_(H1 + P1)	AGGGTGTTTTTATACATCCTGTGAAGTAAAAAAAACCGTA*GTGTAGGCTGGAGCTGCTTC*
SPI-6_T6SS_(H2 + P2)	GTGAACATGGCACATTAATTTGAAGCAGCTCTCATCCGGT*CATATGAATATCCTCCTTAG*
SPI-6_OUT5	CCGAAGTGTATCTGGCGATGA
SD_∆*phoN*_(H1 + P1)	GTGAGTCTTTATGAAAAGTCGTTATTTAGTATTTTTTCTA*GTGTAGGCTGGAGCTGCTTC*
SD_∆*phoN*_(H2 + P2)	ACTTTCACCTTCAGTAATTAAGTTCGGGGTGATCTTCTTT*CATATGAATATCCTCCTTAG*
SD_∆*phoN*_OUT5	TTGCCTGATCCGGAGTGA
K1	CAGTCATAGCCGAATAGCCT
C3	CAGCTGAACGGTCTGGTTATAGG
**VEX Capture**	
SeD_A0289_VEX_H1_U1	TTAACCGGGATCGGGACATGTTCAGCGCAGAAGCAGACTG*GGCCACGTGGGCCGTGCACCTTAAGCTT*
SeD_A0289_VEX_H2_U2	GAGGTTATTCATGTCAACAGGATTACGTTTCACACTGGA*GGTGCAGGCTGGAGCTGCTTC*
SeD_A0326_VEX_H1_D1	GGGGAGGTTGTGCGACGTTTGCATAATCCAGCAAGAACTG*GGTTTAACGGTTGTGGACAACAAGCCAGGG*
SeD_A0326_VEX_H2_D2	ACACAGGCCAGACTGATTATACAGGCATGAAAAAGCTCTC*CAGGTCGACGTCCCATGGCCATTCGAATTC*
SD_VC_OUT5	GCTCTAGACCGGAGGGGTTATCTTTTCC
SD_VC_OUT3	GCTCTAGATTGAAGCAGCTCTCATCCGG
5trfA	ACGTCCTTGTTGACGTGGAAAATGACCTTG
3trfA	CCGGAAGGCATACAGGCAAGAACTGATCG
SPI-6_OUT_DOWN	AAACGGGTCTATTTACAGGGGCAC
**Tiling-PCR**	
1_T6SS_SPI-6_FOR	TTCAAGAAGTTCCACCGTCTATCG
1_T6SS_SPI-6_REV	ACCTGTTTGAGCTGCTACATACCAG
2_T6SS_SPI-6_FOR	CATTCAGTTCGCCGTCAAAGTG
2_T6SS_SPI-6_REV	CCGCTGCGAATTTTGTTATCG
3_T6SS_SPI-6_FOR	CCACGTTCTTCGGCATTACCAG
3_T6SS_SPI-6_REV	CGGTGTTGTAAACCAGATGCTCC
4_T6SS_SPI-6_FOR	AGACGCTGGCGAACACGATC
4_T6SS_SPI-6_REV	TAAGCACTGGCCGTAGCTCTGG
5_T6SS_SPI-6_FOR	GCAGCCATCCTTTGCACAAG
5_T6SS_SPI-6_REV	GGTTGTGTTATTGGCGGCTTC
6_T6SS_SPI-6_FOR	TATGCGATCAGGCGAACCTG
6_T6SS_SPI-6_REV	TCTTCCTGTAACCGGGTATCCAG
7_T6SS_SPI-6_FOR	GGTTGGATCAGGGACTGGATACC
7_T6SS_SPI-6_REV	CGTAACCCTCAACATCCTGCG
8_T6SS_SPI-6_FOR	AAAGCACCGGTGAATGTGGCTG
8_T6SS_SPI-6_REV	TCGGTGTGGTCATCCTTACGGG
9_T6SS_SPI-6_FOR	TGTCAGCACCAACAGTCGCC
9_T6SS_SPI-6_REV	CGCCCTTCGATAGAATCTGGC
10_T6SS_SPI-6_FOR	TAGTAGGGCCAGATTCTATCGAAGG
10_T6SS_SPI-6_REV	CCCTCCGGCTTTTACACATTATTC

^a^Italics indicate the region that anneals to the 5′ or 3′ end of the antibiotic resistance cassette used for the mutagenesis. Underline indicates *Xba*I restriction sites used for cloning an internal region of homology to T6SS_SPI-6_ into R995 plasmid.

### Construction of *Salmonella* Dublin mutant strains

Mutant strains of *Salmonella* Dublin with deletions in the T6SS clusters encoded in SPI-6 (*SeD_A0289* to *SeD_A0326*) and SPI-19 (*SeD_A1212* to *SeD_A1243*) or in the *phoN* gene (*SeD_A4714*) were constructed using the Lambda Red recombination method with modifications [26,27]. The oligonucleotides used for mutagenesis were designed with 40 bases on the 5′ends identical to the ends of the corresponding deletion (Table 2) and 20 bases on the 3′ends that anneal with the 5′or 3′ end of a Cam or Kan resistance cassette flanked by FRT sites (Flp recombinase target sequence) present in plasmids pCLF2 (GenBank accession number HM047089) and pCLF4 (GenBank accession number EU629214.1), respectively. These plasmids were used as templates for the corresponding amplification of PCR products. *Salmonella* Dublin strain CT_02021853 containing the plasmid pKD46, which encodes the Lambda Red recombination system, was grown to an OD_600_ of 0.6 at 30 °C in LB broth supplemented with Amp and L-arabinose (10 mM). Then, bacteria were made electrocompetent and transformed by electroporation with 300 to 600 ng of each PCR product. Transformants were selected on LB agar plates supplemented with the corresponding antibiotic at 37 °C. The presence of each mutation was confirmed by PCR amplification and transferred to the wild-type background by generalized transduction using the high-frequency transducing phage P22 HT105/1 *int*-201.

### Cloning of *Salmonella* Dublin T6SS_SPI-6_ cluster

Cloning of a ~40 Kb fragment encoding the T6SS_SPI-6_ gene cluster from *Salmonella* Dublin CT_02021853 onto plasmid R995 was done by the VEX-Capture system for the targeted excision and cloning of large DNA fragments [28]. In first place, *loxP* sites were introduced at each side of the targeted genomic region by homologous recombination of PCR products by the Lambda-Red system, using as templates the plasmids pVEX1212 and pVEX2212 that encode Sp and Cam resistance cassettes, respectively. The correct insertion of *loxP* sites was confirmed by PCR using primers SPI-6_OUT5 and STM0266_VEX_H2_U2 for *loxP* insertion located in the upstream region of the T6SS cluster, and primers SPI-6_OUT_DOWN and STM0298_VEX_H2_D2 for the downstream *loxP* insertion. T6SS_SPI-6_ cluster was excised from the chromosome as a non-replicating circular DNA molecule by specific recombination of *loxP* sites mediated by the action of Cre recombinase encoded in plasmid pEKA30. A 1,209 bp internal region of SPI-6 was amplified using primers SD_VC_OUT5 and SD_VC_OUT3, both of which include an *Xba*I restriction site at the 5′ end. The PCR product was cloned into the unique *Xba*I site in R995 to generate R995-VC6 (Table 2). The T6SS_SPI-6_ intermediate was then captured into the R995-VC6 vector by a homologous recombination event, producing the R995 + SPI-6 plasmid.

Plasmid R995 + SPI-6 was transferred to *E. coli* strain EC100D *pir-116* by conjugation and the presence and structural integrity of the T6SS_SPI-6_ gene cluster cloned onto R995 was verified by visualization of supercoiled plasmid DNA in agarose gel and by tiling-PCR analysis which amplify ten fragments that cover the entire T6SS region. *E. coli* strains EC100D *pir-116*/ R995 + SPI-6 and R995 + SPI-19 were used as donors for transfer of the captured SPI-6 and SPI-19 to the *Salmonella* Dublin ∆T6SS_SPI-6_/∆T6SS_SPI-19_ strain by conjugation.

For competitive infections in chickens and mice, the in vivo stability of plasmids R995 and R995 + SPI-6 was assessed in each organ at each time point studied. No differences were observed on colony forming units (CFU) indicating that R995 and its derivatives are highly stable in vivo.

### Animal infections

#### Ethics statement

All animal experiments conducted in this study were approved by the Texas A&M University Institutional Animal Care and Use Committee (TAMU AUP# 2010–38) and were carried out in accordance with the Guide to the Care and Use of Laboratory Animals, the Public Health Service Policy on the Human Care and Use of Laboratory Animals.

#### Chicken experiments

For competitive infections in the avian model, fifteen 4-day-old unsexed White Leghorn chicks were orally inoculated with 10^9^ CFU of an equal mixture of the strains to be tested in a volume of 100 μL of sterile PBS. The exact titer and ratio of strains in the inoculum were determined by serial dilution and plating on LB agar supplemented with the corresponding antibiotics. Five birds from the infected group were sacrificed by asphyxiation with CO_2_ on days 1, 3 and 9 post-infection. Cecum (with contents), liver and spleen were collected and homogenized in sterile PBS. Then, serial ten-fold dilutions were spread on LB agar plates containing the appropriate antibiotics for determination of CFU.

#### Mouse experiments

For competitive infections in the murine model, groups of five six- to eight-week-old female BALB/c mice were inoculated with 10^6^ CFU of an equal mixture of the strains to be tested in a volume of 100 μL sterile PBS. The exact titer and ratio of strains in the inoculum were determined as described above. Four days post-infection, mice were sacrificed and cecum, liver and spleen were collected and homogenized. The number of Salmonellae present in each organ were enumerated as described above. In both animal models, the *Salmonella* Dublin ∆*phoN* mutant was used as wild-type strain. Inactivation of *phoN*, encoding alkaline phosphatase, abolishes the ability to cleave 5-bromo-4-chloro-3-indolyl phosphate (XP), but does not reduce the ability of *Salmonella* to colonize chicken and mice [21,29]. Growth on Luria–Bertani (LB) agar plates supplemented with XP provided an easy means to distinguish between the wild-type strain (PhoN-, white colonies) and T6SS mutant strains (PhoN+, blue colonies) in competitive infection experiments.

### Data analysis

CFU obtained from competitive experiments were used for data analysis as a mean ratio of logarithmically converted CFU of mutant to wild type, normalized to the input ratio. Error bars indicate standard error. A parametric test (Student’s *t*-test) was used to determine whether differences between treatment groups were statistically significant (*P* < 0.05).

## Results

### Role of the T6SSs encoded in SPI-6 and SPI-19 to *Salmonella* Dublin colonization of mice

*Salmonella* Dublin contains two phylogenetically distinct T6SSs (T6SS_SPI-6_ and T6SS_SPI-19_), that have been individually linked to virulence in other *Salmonella* serotypes [20,22-25]. To determine if either one or both T6SSs contribute to colonization of the murine host by *Salmonella* Dublin, we first performed competitive infections between a mutant carrying deletions of both T6SS_SPI-6_ and T6SS_SPI-19_ gene clusters (∆T6SS_SPI-6_/∆T6SS_SPI-19_) and the wild-type strain of *Salmonella* Dublin.

**Figure 1:**
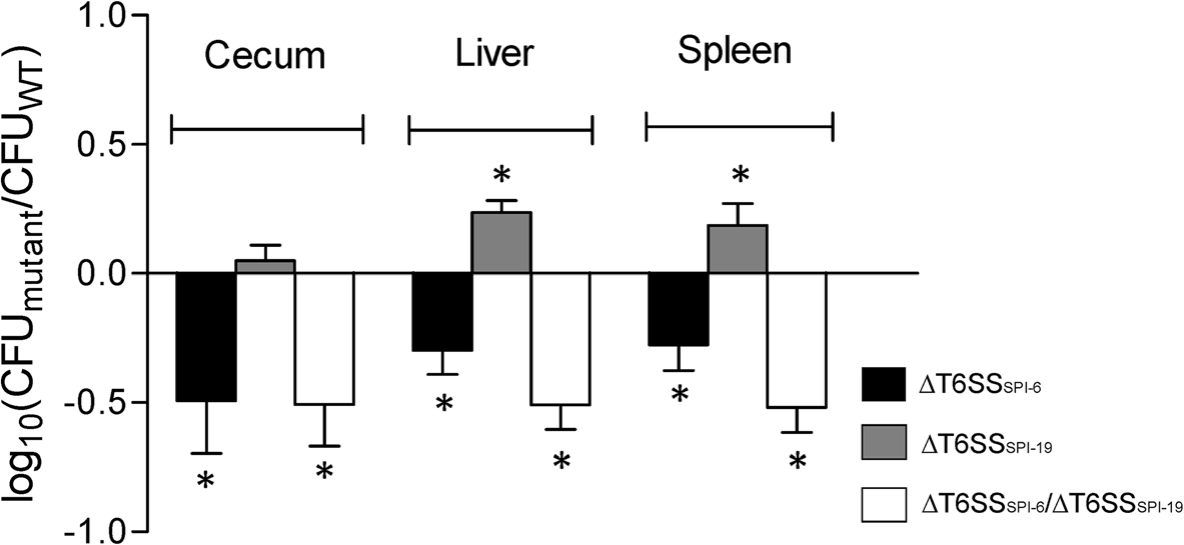
**In vivo competition experiments between ∆T6SS**_**SPI-6**_**/∆T6SS**_**SPI-19**_**, ∆T6SS**_**SPI-6 **_**and ∆T6SS**_**SPI-19 **_**deletion mutants and wild type *Salmonella *Dublin strain CT_02021853 in orally infected mice.** Five six to eight-weeks-old female BALB/c mice were infected orally with 10^6^ CFU of a 1:1 mixture of the corresponding T6SS mutant strain and wild type *Salmonella* Dublin CT_02021853. After 4 days of infection, mice were eutanized and the cecum, liver and spleen were aseptically removed and homogenized in sterile PBS. Bacterial load recovered from each organ was determined by plating serial ten-fold dilutions on LB agar plates with the appropriate antibiotics. Bars represent mean values ± standard error. Statistical significance was calculated using the Student’s *t* test. Asterisks indicate the statistical significance of differences between the normalized output ratio and the equivalent ratio in the inoculum. * *P* value < 0.05.

As shown in Figure 1, the ∆T6SS_SPI-6_/∆T6SS_SPI-19_ double mutant showed a statistically significant colonization defect in each organ tested. To determine the individual contribution of each T6SS to this phenotype, competitive infections were performed between the wild-type strain and the corresponding single ∆T6SS_SPI-6_ and ∆T6SS_SPI-19_ mutant. As observed in Figure 1, only the ∆T6SS_SPI-6_ mutant strain was attenuated, displaying a colonization defect very similar to the ∆T6SS_SPI-6_/∆T6SS_SPI-19_ double mutant. In contrast, the ∆T6SS_SPI-19_ mutant reached the same levels of colonization as the wild type strain in all organs analyzed, suggesting that only T6SS_SPI-6_ is involved in mice colonization.

**Figure 2:**
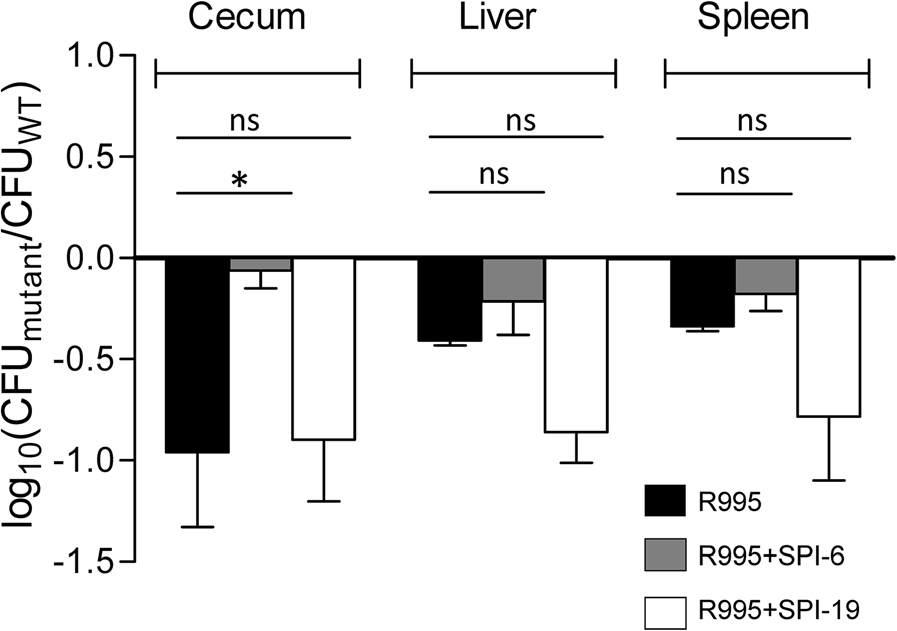
**In vivo competition experiment between ∆T6SS**_**SPI-6**_**/∆T6SS**_**SPI-19 **_**mutant complemented *in trans *with T6SS**_**SPI-6 **_**or T6SS**_**SPI-19 **_**and wild type *Salmonella *Dublin strain CT_02021853 in orally infected mice.** Fifteen six to eight-weeks-old female BALB/c mice were orally infected with 10^6^ CFU of a 1:1 mixture of strains WT/R995, (∆T6SS_SPI-6_/∆T6SS_SPI-19_)/R995 + SPI-6 and (∆T6SS_SPI-6_/∆T6SS_SPI-19_)/R995 + SPI-19. After 4 days of infection, mice were eutanized and the cecum, liver and spleen were aseptically removed and homogenized in sterile PBS. Bacterial load recovered from each organ was determined by plating serial ten-fold dilutions on LB agar plates with the appropriate antibiotics. Bars represent mean values ± standard error. Statistical significance was calculated using the Student’s *t* test. Asterisks indicate the statistical significance of differences between data sets. * *P* value < 0.05; ns, not significant.

To verify that only T6SS_SPI-6_ is responsible for the colonization defect observed during mice infection, the double mutant was complemented *in trans* with either T6SS_SPI-6_ (R995 + SPI-6) or T6SS_SPI-19_ (R995 + SPI-19) and competition experiments were performed. As shown in Figure 2, transfer of T6SS_SPI-6_ restored the ability of the ∆T6SS_SPI-6_/∆T6SS_SPI-19_ double mutant to colonize the cecum. However, complementation was not achieved in the liver and spleen (Figure 2). Interestingly, transfer of T6SS_SPI-19_ did not restore the colonization defect of the ∆T6SS_SPI-6_/∆T6SS_SPI-19_ mutant strain in all organs, indicating that only T6SS_SPI-6_ is involved in colonization of mice by *Salmonella* Dublin.

### Contribution of the T6SSs encoded in SPI-6 and SPI-19 to *Salmonella* Dublin colonization of the avian host

**Figure 3:**
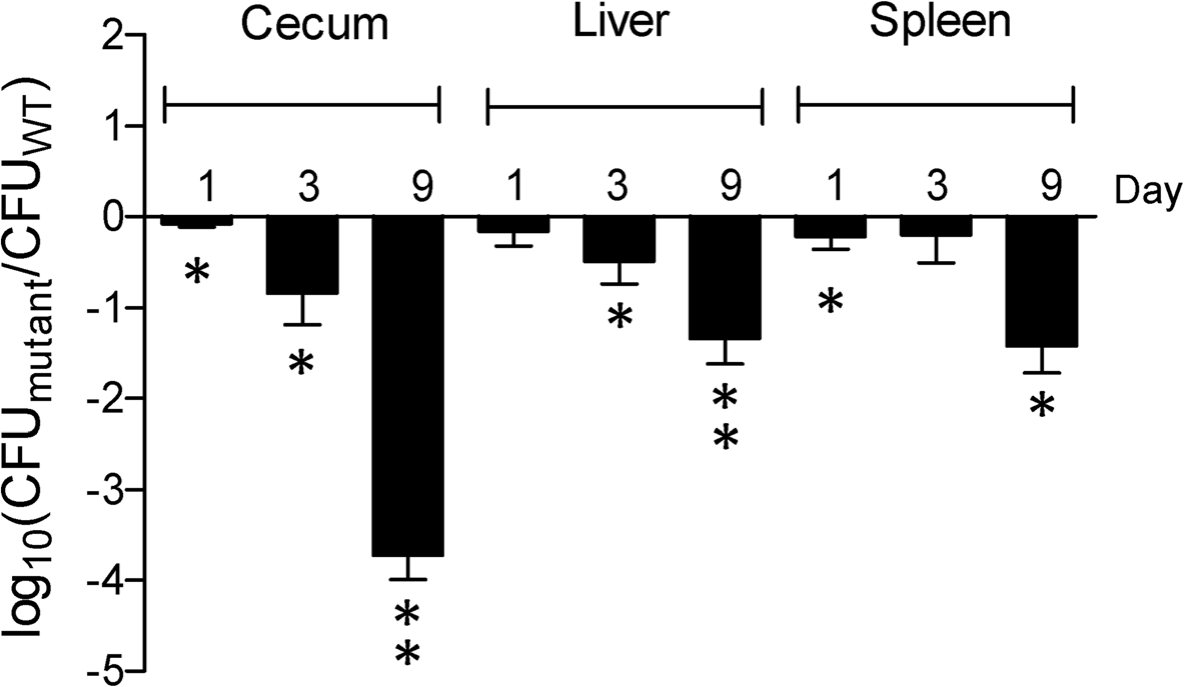
**In vivo competition experiment between ∆T6SS**_**SPI-6**_**/∆T6SS**_**SPI-19 **_**deletion mutant and wild type *Salmonella *Dublin strain CT_02021853 in orally infected chicks.** Fifteen four-day-old White Leghorn chicks were infected intragastrically with 10^9^ CFU of a 1:1 mixture of the mutant strain and wild type *Salmonella* Dublin CT_02021853. At 1, 3 and 9 days after the inoculation, groups of five chicks were sacrificed and the cecum, liver and spleen were aseptically excised and homogenized in sterile PBS. Bacterial load recovered from each organ was determined by plating serial ten-fold dilutions on LB agar plates with the appropriate antibiotics. Bars represent mean values ± standard error. Statistical significance was calculated using the Student’s *t* test. Asterisks indicate the statistical significance of differences between the normalized output ratio and the equivalent ratio in the inoculum. ** *P* value < 0.001; * *P* value < 0.05.

To determine the contribution of T6SS_SPI-6_ and T6SS_SPI-19_ to colonization of chicks by *Salmonella* Dublin, competitive infections were performed between the (∆T6SS_SPI-6_/∆T6SS_SPI-19_) mutant and the wild-type strain. As shown in Figure 3, the ∆T6SS_SPI-6_/∆T6SS_SPI-19_ double mutant was defective for both intestinal and systemic colonization early after infection (days 1 and 3 post-infection). The colonization defect was most severe by day 9 post-infection in all organs, especially in the cecum. In contrast to the results obtained in the murine model, this mutant showed a very strong colonization defect in cecum, while only a mild phenotype was observed in the liver and spleen, indicating a critical role for the T6SSs to gastrointestinal colonization of the chicken by *Salmonella* Dublin.

**Figure 4:**
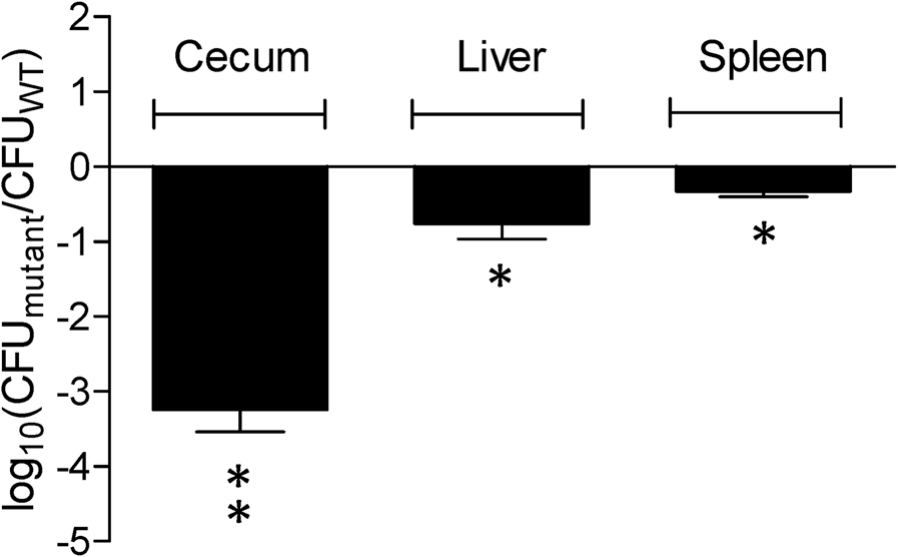
**In vivo competition between ∆T6SS**_**SPI-6 **_**deletion mutant and the wild type *Salmonella *Dublin strain CT_02021853 in orally infected chicks.** Five four-day-old White Leghorn chicks were infected intragastrically by gavage with 10^9^ CFU of a mixture at a 1:1 ratio of the ∆T6SS_SPI-6_ mutant strain and the wild type *Salmonella* Dublin CT_02021853. At day 9 post-infection chicks were sacrificed and organs were excised, homogenized, and serially diluted to determine bacterial loads. Bars represent the geometric mean of the log ratio of the mutant CFU/wild type CFU, normalized to the inoculum ratio. Error bars denote standard error. Statistical significance was determined using a two-tailed Student’s *t* test, and asterisks indicate that normalized output ratios were significantly statistically different from the equivalent ratio in the inoculum (* *P* < 0.05; ** *P* < 0.001).

Competitive index experiments performed at day 9 post-infection showed that a T6SS_SPI-6_ mutant strain was defective for chicken colonization to the same extent as the ∆T6SS_SPI-6_/∆T6SS_SPI-19_ double mutant strain, suggesting that the T6SS encoded in SPI-6 is crucial for an efficient colonization of the avian host (Figure 4).

**Figure 5:**
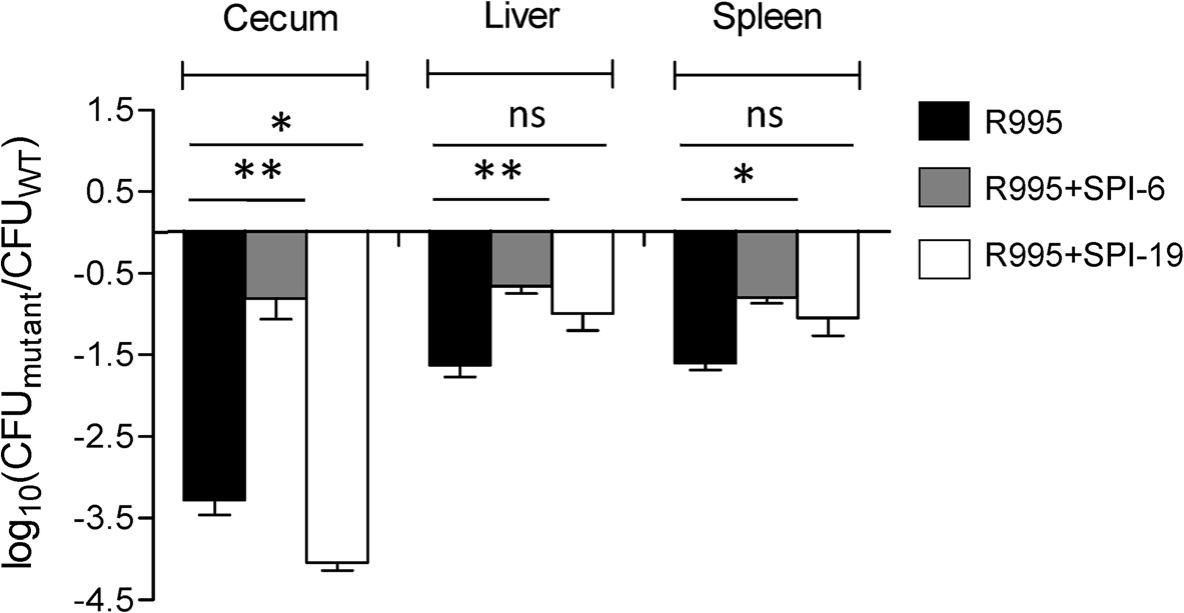
**In vivo competition experiment between ∆T6SS**_**SPI-6**_**/∆T6SS**_**SPI-19 **_**mutant complemented *in trans *with T6SS**_**SPI-6 **_**or T6SS**_**SPI-19 **_**and wild type *Salmonella *Dublin strain CT_02021853 in orally infected chicks.** Fifteen four-day-old White Leghorn chicks were orally infected with 10^9^ CFU of a 1:1 mixture of strains WT/R995, (∆T6SS_SPI-6_/∆T6SS_SPI-19_)/R995 + SPI-6 and (∆T6SS_SPI-6_/∆T6SS_SPI-19_)/R995 + SPI-19. At 1, 3 and 9 days post-infection, groups of five chicks were sacrificed and the cecum, liver and spleen were aseptically excised and homogenized in sterile PBS. Bacterial load recovered from each organ was determined by plating serial ten-fold dilutions on LB agar plates with the appropriate antibiotics. Bars represent mean values ± standard error. Statistical significance was calculated using the Student’s *t* test. Asterisks indicate the statistical significance of differences between data sets. ** *P* value < 0.001; * *P* value < 0.05.

To verify that T6SS_SPI-6_ was responsible for the phenotypes observed during chicken infection, the ∆T6SS_SPI-6_/∆T6SS_SPI-19_ deletion mutant was complemented *in trans* with either T6SS_SPI-6_ (R995 + SPI-6) or T6SS_SPI-19_ (R995 + SPI-19) and competition experiments were performed at day 9 post-infection. As shown in Figure 5, transfer of T6SS_SPI-6_, but not T6SS_SPI-19_, complemented the colonization defect of the ∆T6SS_SPI-6_/∆T6SS_SPI-19_ double mutant in each organ tested, indicating that T6SS_SPI-6_ was responsible for the colonization defect of the double mutant strain. Altogether our data shows that only the T6SS_SPI-6_ contributes to colonization of the murine and avian host.

## Discussion

The presence of multiple T6SSs has been reported in several bacterial species suggesting functional adaptation of each T6SS to a specific niche and/or host. For example, of the five T6SS (T6SS-1 to T6SS-5) of *Burkholderia thailandesis,* T6SS-5 is important for virulence while T6SS-1 participates in killing of susceptible target bacteria [15]. This is also the case for *Pseudomonas aeruginosa*, which encodes three T6SS loci (HSI-I to HSI-III).HSI-I mediates inter-bacterial relationships [17], while HSI-II and HSI-III are required for virulence towards animals and plants [30].

*Salmonella* Dublin encodes two T6SSs (T6SS_SPI-6_ and T6SS_SPI-19_) that have been individually linked to virulence and pathogenesis in other *Salmonella* serotypes. Notably, while most *Salmonella* serotypes seem to have lost the T6SS_SPI-6_ locus after acquisition of SPI-19, *Salmonella* Dublin has retained both T6SSs. Whether the presence of these two T6SS corresponds to differential functional specialization of each system or whether they are redundant in their function, is currently unknown.

In this study we performed competitive infection experiments to determine the contribution of both T6SS to colonization of chickens and mice by *Salmonella* Dublin, and to gain insights into the potential functional adaptation of T6SS_SPI-6_ and T6SS_SPI-19_ to either animal host. We chose the murine and avian models of infection because it has been reported that *Salmonella* Dublin asymptomatically colonizes mice and chickens, suggesting that these animals can act as reservoirs and vectors for *Salmonella* Dublin infection in cattle and humans [11,12] and because previous studies have individually linked the T6SS_SPI-6_ and T6SS_SPI-19_ of other serotypes to the ability of *Salmonella* to colonize the murine and avian host.

Competitive index data from oral infection of White Leghorn chicks and BALB/c mice showed that a mutant strain lacking both T6SS_SPI-6_ and T6SS_SPI-19_ was not able to colonize the cecum, liver and spleen as efficiently as the wild-type strain in both animal models. Interestingly, even though the double mutant was attenuated in chickens and mice, the degree of attenuation was different depending on the infected animal host. Thus, while the double mutant strain was only slightly attenuated in all organs in mice (log competitive index of -0.5), it was severely attenuated in the chicken, displaying a log competitive index of -3.72 in the cecum and of ~ -1.5 in the liver and spleen. Interestingly, a similar phenotype was observed for a single T6SS_SPI-6_ mutant strain in both models of infection.

The strong colonization defect observed in the cecum of infected chicks was not unexpected, as previous studies performed in *Salmonella* Typhimurium have shown that T6SS_SPI-6_ is required for efficient colonization of the cecum of infected chicks [21]. In agreement with this, our complementation experiments demonstrated that T6SS_SPI-6_ was responsible for these phenotypes, as transfer of the captured T6SS_SPI-6_ gene cluster, but not of the T6SS_SPI-19_ gene cluster, restored the ability of the double mutant to colonize the gastrointestinal tract and internal organs of infected chickens and mice.

Our data strongly suggests that *Salmonella* Dublin requires a functional T6SS_SPI-6_ for efficient colonization and persistence in the avian gastrointestinal tract and that T6SS_SPI-19_ is not involved in this process. These differences support the notion that T6SS_SPI-6_ and T6SS_SPI-19_ are not functionally redundant. The fact that T6SS_SPI-19_ is important for colonization of the avian host by *Salmonella* Gallinarum [25], but not by *Salmonella* Dublin suggests that the contribution and impact of the T6SSs to *Salmonella* pathogenesis depend on the serotype and the infected host.

The wide distribution of the T6SS_SPI-6_ among *Salmonella enterica* serotypes [18] and the fact that this T6SS has been shown to be required for host-colonization in each serotype tested [20-23] suggests that this T6SS is part of the common virulence gene pool of *Salmonella enterica*. This would not be the case for the T6SS_SPI-19_ which has a much limited distribution and, as shown by this study, is not important for host-colonization in all serotypes.

The mechanisms behind the contribution of the T6SS_SPI-6_ to *Salmonella* Dublin virulence remain obscure. Previous reports have shown that, in *Salmonella* Typhimurium and *Salmonella* Typhi, the T6SS_SPI-6_ contributes to *Salmonella* survival within murine and avian macrophages [20,22], nevertheless we could not detect a significant contribution of the T6SS_SPI-6_ of *Salmonella* Dublin to these processes (data not shown, Bernardo Pinto MSc. Thesis).

The question of why *Salmonella* Dublin has retained both T6SSs is still unanswered. Nevertheless, we cannot rule out that T6SS_SPI-19_ may contribute to *Salmonella* fitness in other natural settings, such as the environment, or to colonization of other animals such as cattle, which is the natural host for *Salmonella* Dublin. It is possible that T6SS_SPI-19_ might play a role in the course of systemic diseases such as typhoid caused by *Salmonella* Dublin and *Salmonella* Gallinarum in cattle and chickens, respectively. Further studies will have to be conducted to evaluate the contribution of both T6SS_SPI-6_ and T6SS_SPI-19_ to the ability of *Salmonella* Dublin to colonize cattle.

Altogether, our data shows that T6SS_SPI-6_ contributes to chicken and mice colonization by *Salmonella* Dublin and that T6SS_SPI-19_ is not involved in these processes. The ancestral acquisition of the T6SS_SPI-6_ locus, its wide distribution among *S. enterica* serotypes and its contribution to virulence in *Salmonella* Dublin, *Salmonella* Typhimurium and *Salmonella* Typhi suggest that this T6SS belongs to the common tool-box used by *S. enterica* to infect and colonize a wide variety of animal hosts.

## Competing interests

The authors declare that they have no competing interests.

## Authors’ contributions

Conceived and designed the experiments: DP, CJB, HAP, CAS, IC. Performed the experiments: DP, HJY, CJB. Analyzed the data: DP, CJB, CAS, HAP, IC. Contributed reagents/materials/analysis tools: HAP, CAS, IC. Wrote the paper: DP, CJB, HAP, CAS, IC. All authors read and approved the final manuscript.
